# Impact of clinically diagnosed liver cirrhosis in patients with intrahepatic cholangiocarcinoma treated with systemic chemotherapy: a subgroup analysis of JCOG1113

**DOI:** 10.1093/jjco/hyaf035

**Published:** 2025-03-07

**Authors:** Mao Okada, Eiichiro Suzuki, Chigusa Morizane, Gakuto Ogawa, Yusuke Sano, Hiroshi Imaoka, Satoshi Kobayashi, Masafumi Ikeda, Naohiro Okano, Haruo Miwa, Akiko Todaka, Satoshi Shimizu, Nobumasa Mizuno, Sohei Satoi, Keiji Sano, Kazutoshi Tobimatsu, Akio Katanuma, Kenkichi Masutomi, Takuji Okusaka, Masato Ozaka, Makoto Ueno, the members of the Hepatobiliary and Pancreatic Oncology Group of the Japan Clinical Oncology Group (JCOG-HBPOG)

**Affiliations:** Department of Hepatobiliary and Pancreatic Oncology, National Cancer Center Hospital, 5-1-1 Tsukiji, Chuo-ku, Tokyo 104-0045, Japan; Courses of Advanced Clinical Research of Cancer, Juntendo University Graduate School of Medicine, 2-1-1, Hongo, Bunkyo-ku, Tokyo 113-8421, Japan; Department of Gastroenterology, Graduate School of Medicine, Chiba University, Chiba, 1-8-1 Inohana, Chuo-ku, Chiba-shi, Chiba 260-8670, Japan; Department of Hepatobiliary and Pancreatic Oncology, National Cancer Center Hospital, 5-1-1 Tsukiji, Chuo-ku, Tokyo 104-0045, Japan; Japan Clinical Oncology Group Data Center/Operations Office, National Cancer Center Hospital, 5-1-1 Tsukiji, Chuo-ku, Tokyo 104-0045, Japan; Japan Clinical Oncology Group Data Center/Operations Office, National Cancer Center Hospital, 5-1-1 Tsukiji, Chuo-ku, Tokyo 104-0045, Japan; Department of Hepatobiliary and Pancreatic Oncology, National Cancer Center Hospital East, 6-5-1 Kashiwanoha, Kashiwa, Chiba 277-8577, Japan; Department of Gastroenterology, Hepatobiliary and Pancreatic Medical Oncology Division, Kanagawa Cancer Center, 2-3-2 Nakao, Asahi-ku, Yokohama, Kanagawa 241-8515, Japan; Department of Hepatobiliary and Pancreatic Oncology, National Cancer Center Hospital East, 6-5-1 Kashiwanoha, Kashiwa, Chiba 277-8577, Japan; Department of Medical Oncology, Kyorin University Faculty of Medicine, 6-20-2 Shinkawa, Mitaka-shi, Tokyo 181-8611, Japan; Gastroenterological Center, Yokohama City University Medical Center, 4-57 Urafune-cho, Minami-ku, Yokohama-shi, Kanagawa 232-0024, Japan; Division of Gastrointestinal Oncology, Shizuoka Cancer Center, 1007 Shimo-Nagakubo, Nagaizumi-Cho, Sunto-Gun, Shizuoka 411-8777, Japan; Department of Medical Oncology and Hematology, Oita University Faculty of Medicine, 1-1 Idaigaoka, Hasama-machi, Yufu-city, Oita 879-5593, Japan; Department of Gastroenterology, Saitama Cancer Center, 780 Komuro, Ina-machi, Kitaadati-gun, Saitama 362-0806, Japan; Department of Gastroenterology, Aichi Cancer Center Hospital, 1-1 Kanokodono, Chikusa-ku, Nagoya 464-8681, Japan; Department of Surgery, Kansai Medical University Hospital, 3-1 Shinmachi 2 Chome, Hirakata City, Osaka 573-1191, Japan; Department of Surgery, Teikyo University School of Medicine, 2-11-1 Kaga, Itabashi-Ku, Tokyo 173-8606, Japan; Division of Gastroenterology, Department of Internal Medicine, Kobe University Graduate School of Medicine, 7-5-1 Kusunoki-cho, Chuo-ku, Kobe City, Hyogo, 650-0017, Japan; Center for Gastroenterology, Teine Keijinkai Hospital, 1-40, 12-chome, Maeda 1-jo, Teine-ku, Sapporo-shi, Hokkaido, Japan; Courses of Advanced Clinical Research of Cancer, Juntendo University Graduate School of Medicine, 2-1-1, Hongo, Bunkyo-ku, Tokyo 113-8421, Japan; Division of Cancer Stem Cell, National Cancer Center Research Institute, 5-1-1 Tsukiji, Chuo-ku, Tokyo 104-0045, Japan; Department of Hepatobiliary and Pancreatic Oncology, National Cancer Center Hospital, 5-1-1 Tsukiji, Chuo-ku, Tokyo 104-0045, Japan; Hepato-Biliary-Pancreatic Chemotherapy Division, Cancer Institute Hospital of Japanese Foundation for Cancer Research, 3-8-31 Ariake, Koto-ku, Tokyo, 135-8550, Japan; Department of Gastroenterology, Hepatobiliary and Pancreatic Medical Oncology Division, Kanagawa Cancer Center, 2-3-2 Nakao, Asahi-ku, Yokohama, Kanagawa 241-8515, Japan

**Keywords:** intrahepatic cholangiocarcinoma, liver cirrhosis, chemotherapy

## Abstract

**Background:**

The JCOG1113, a multicenter, randomized phase III trial in patients with advanced/recurrent biliary tract cancer showed the non-inferiority of gemcitabine plus S-1 to gemcitabine plus cisplatin. Although liver cirrhosis (LC) is a known risk factor for intrahepatic cholangiocarcinoma (ICC), few reports focus on the efficacy and safety of chemotherapy in ICC patients with LC.

**Methods:**

We performed a subgroup analysis of ICC patients enrolled in the JCOG1113. The presence or absence of LC was evaluated based on clinical factors such as radiographic findings, medical history, laboratory data, and physical examination at enrollment. We evaluated differences in the safety and efficacy of chemotherapy according to the presence or absence of clinically diagnosed LC.

**Results:**

Of the 94 eligible patients with ICC, 10 were clinically diagnosed with LC. In the non-LC/clinically diagnosed LC group, grade 3 or 4 neutropenia, anemia, decreased platelet count, and non-hematological adverse events were observed in 51.2%/60%, 15.5%/0%, 11.9%/40%, and 38.1%/30% patients. The median overall survival was 13.7 months in the non-LC group and 19.0 months in the clinically diagnosed LC group (hazard ratio [HR]: 0.969, 95% confidence interval [CI]: 0.482–1.948). The median progression-free survival was 5.9 months in the non-LC group and 7.1 months in the clinically diagnosed LC group (HR, 0.995; 95% CI, 0.513–1.929).

**Conclusion:**

The results of this study indicated that eligible ICC patients with clinically diagnosed LC, as determined by clinical and CT imaging findings, did not exhibit any apparent safety or efficacy disadvantage compared to those without LC.

## Teaser

The results of this subgroup analysis of JCOG1113 showed no apparent safety or efficacy disadvantage of clinically diagnosed LC in patients with ICC treated with systemic chemotherapy.

## Introduction

Biliary tract cancer (BTC) is one of the cancers with a poor prognosis and is often diagnosed at an advanced stage. Intrahepatic cholangiocarcinoma (ICC) is classified as a primary liver cancer according to the Union for International Cancer Control (UICC) classification, but systemic chemotherapy for advanced or recurrent cases is administered according to BTC. The majority of primary liver cancers are hepatocellular carcinoma (HCC) (75%–85%), and ICC has been reported to be 10%–15% [1]. Risk factors for ICC are reported to include hepatitis B (HBV) [2] and hepatitis C (HCV) [2,3], liver cirrhosis (LC) from any cause, primary sclerosing cholangitis, liver flukes, metabolic syndrome, and alcohol intake [2–9]. According to a follow-up survey report by the Liver Cancer Study Group in Japan [10], 5.8% of the patients with ICC were positive for the HBs antigen, and 12.8% were positive for the HCV virus antibody.

Gemcitabine plus cisplatin (GC) is one of the standard international treatment regimens for advanced or recurrent BTC [11]. JCOG1113, a randomized phase III trial conducted by the Japan Clinical Oncology Group (JCOG) Hepatobiliary and Pancreatic Oncology Study Group in Japan, confirmed that gemcitabine plus S-1 (GS) is non-inferior to GC as first-line chemotherapy for unresectable or recurrent BTC including ICC, extrahepatic cholangiocarcinoma, gallbladder cancer, and ampulla of Vater. JCOG1113 enrolled 354 patients from May 2013 to March 2016 and showed that GS was non-inferior to GC in overall survival (OS) (median OS, GC 13.4 months, GS 15.1 months; hazard ratio [HR] 0.945; 90% confidence interval [CI] 0.78**–**1.15; *P* = .046 for non-inferiority) and was well tolerated [12]. The results of the JCOG1113 have made GS a treatment option for chemotherapy in unresectable or recurrent BTC.

Some anticancer drugs used in BTC, such as gemcitabine and S-1, are metabolized in the liver. With regard to the application of these anticancer drugs in patients with LC, adverse events (AEs) might increase due to impaired drug metabolism from hepatic dysfunction [13–15]. In addition, there are concerns regarding the severity of myelosuppression due to pancytopenia induced by LC [16], and anticancer drugs can cause liver damage [17]. Several previous reports have considered whether the dosage of anticancer drugs should be reduced in patients with liver dysfunction [18–20]. However, few clinical reports have elucidated such concerns or evaluated the safety and efficacy of systemic chemotherapy in patients with ICC having LC. Thus, it is important to assess the effect of LC on chemotherapy safety in patients with ICC.

In the present study, we analyzed the safety and efficacy of systemic chemotherapy in patients with ICC enrolled in JCOG1113 [12] by the presence or absence of clinically diagnosed LC.

## Patients and methods

### Study setting of JCOG1113

The main eligibility criteria of JCOG1113 were as follows: histologically proven BTC, unresectable or recurrent disease, age 20–79 years, Eastern Cooperative Oncology Group performance status score 0 or 1, ability to take oral intake, no prior therapy for BTC except for surgery or biliary drainage, no prior chemotherapy or radiation therapy, and adequate function of major organs such as platelet count >100 × 10 [9]/l, aspartate aminotransferase and alanine aminotransferase concentration ≤ 100 U/L (150 U/l in biliary drainage cases), and total bilirubin ≤2.0 mg/dl (3.0 mg/dl in biliary drainage cases) [12]. The GC arm received gemcitabine (1000 mg/m^2^) and cisplatin (25 mg/m^2^) on days 1 and 8, which was repeated every 3 weeks. Cisplatin was administered up to 16 times (400 mg/m^2^) unless the patient met the termination criteria. In the GS arm, gemcitabine (1000 mg/m^2^) was administered on days 1 and 8, and S-1 was administered orally twice daily [60 mg/day for body surface area (BSA) < 1.25 m^2^, 80 mg/day for BSA 1.25–1.50 m^2^, and 100 mg/day for BSA ≥ 1.50 m^2^] on days 1–14 and repeated every 3 weeks. In both arms, the protocol treatment was continued until disease progression, unacceptable toxicity, or patient refusal was observed [14]. Written informed consent was obtained at the time of registration for the JCOG1113. The study protocol was approved by the institutional review board of each participating institution.

The subgroup analyses complied with the ethical standards of the World Medical Association (Declaration of Helsinki). The JCOG1113 was registered at UMIN Clinical Trials Registry, number UMIN000010667.

### Design of this subgroup analysis

This was a subgroup analysis of the JCOG1113. We used the clinical information and laboratory data at the time of enrollment in the JCOG1113.

Although a definitive diagnosis of LC requires a pathological diagnosis, the JCOG1113 did not obtain pathological information on non-tumor liver tissues. Therefore, LC-related clinical factors were obtained based on radiological findings, clinical history, laboratory data, and physical findings at the time of enrollment. Radiological findings were evaluated based on factors [21,22] such as morphological changes (atrophy or enlargement considered characteristic of LC) in the liver, except for changes caused by the tumor, coarsening of the liver parenchyma, blunting of the liver edge, irregular surface of the liver, and development of hepatic collateral blood vessels, splenomegaly, varices, or ascites. The diagnosis of LC by imaging was made by individual local physicians. Since a diagnostic radiology central review was not conducted in JCOG1113, the present subgroup analysis also did not include a diagnostic radiology central review of LC, as it relied on clinical information collected in the primary study. Based on these data, the physician comprehensively determined whether each patient had clinically diagnosed LC and noted it in the case report forms.

The Child–Pugh score was originally developed to assess the prognosis of patients with LC and portal hypertension undergoing surgery for variceal bleeding [23,24]. It is widely used to assess the liver function in patients with LC. The score was not intended to evaluate liver function in non-LC patients or distinguish between LC and non-LC patients; however, in this study, the Child–Pugh score was also assessed in non-LC patients for the purpose of simplified and consistent evaluation of liver function as a reference data.

### Endpoints and statistical analysis

This study evaluated the percentage of planned doses and number of courses administered, AEs, and efficacy. AEs were reported according to the Common Terminology Criteria for Adverse Events (CTCAE) version 4.0 in the JCOG1113. Regarding the evaluation of efficacy, OS was defined as the time from the date of study enrollment to the date of death from any cause or the last follow-up date. Progression-free survival (PFS) was defined as the time from the date of study enrollment to the date of documented disease progression or death. The response rate (RR) was analyzed in patients with measurable lesions. It was defined as the percentage of patients with a complete or partial response according to the Response Evaluation Criteria in Solid Tumors version 1.1.

OS and PFS were estimated using the Kaplan–Meier method. HRs and corresponding 95% CIs were estimated using the Cox regression hazard model. Statistical analyses were performed using SAS software version 9.4. All statistical analyses were performed at the JCOG Data Center.

## Results

### Patients

Ninety-four patients with ICC were enrolled in the JCOG1113, and all were included in this analysis. Of the 94 patients, 84 were classified as non-LC, and 10 were clinically diagnosed with LC (Table 1). Patient backgrounds are shown in Table 2. The proportion of patients with a history of heavy alcohol consumption (average daily intake of ≥60 g of pure ethanol) was higher in the clinically diagnosed LC group than in those without (20.0% vs. 11.9%). Regarding the hepatitis virus, no patients were positive for HBV (HBs antigen or HBV DNA) or HCV antibody in the clinically diagnosed LC group, whereas 2.4% were HBV-positive, and 3.6% were HCV antibody-positive in the non-LC group. The number of patients with Child–Pugh score ≥8 was 9 (10.7%) in non-LC group and 1 (10.0%) in the clinically diagnosed LC group. The number of patients with a history of biliary drainage was 23 (27.4%) in the non-LC group and one (10.0%) in the clinically diagnosed LC group. The proportions of patients treated with GC/GS were well balanced (53.6% and 46.4% in the non-LC group and 50% and 50% in the clinically diagnosed LC group, respectively).

**Table 1:** Factors related to the diagnosis of liver cirrhosis (*n* = 94)

CT findings characteristic of liver cirrhosis	*n* (%)
Morphological change Absent Present	53 (56.4) 41 (43.6)
Parenchymal coarsening Absent Present	88 (93.6) 6 (6.4)
Blunt edge Absent Present	74 (78.7) 20 (21.3)
Irregular surface Absent Present	86 (91.5) 8 (8.5)
Varices, collaterals or splenomegaly Absent Present	81 (86.2) 13 (13.8)
Ascites Absent Present	75 (79.8) 19 (20.2)
Clinical diagnosis of liver cirrhosis No Yes	84 (89.4) 10 (10.6)

CT, computed tomography.

**Table 2:** Patient backgrounds

	Non-LC			Clinically diagnosed LC			Total
	GC	GS	Total	GC	GS	Total	*n* = 94
	*n* = 45	*n* = 39	*n* = 84	*n* = 5	*n* = 5	*n* = 10	
Age							
Median	67	66	67	66	68	67	67
Range	50–77	45–78	45–78	56–72	46–73	46–73	45–78
Sex							
Male (%)	26 (57.8)	22 (56.4)	48 (57.1)	5 (100.0)	3 (60.0)	8 (80.0)	56 (59.6)
Female (%)	19 (42.2)	17 (43.6)	36 (42.9)	0 (0.0)	2 (40.0)	2 (20.0)	38 (40.4)
PS							
0	32 (71.1)	24 (61.5)	56 (66.7)	4 (80.0)	5 (100.0)	9 (90.0)	65 (69.1)
1	13 (28.9)	15 (38.5)	28 (33.3)	1 (20.0)	0 (0.0)	1 (10.0)	29 (30.9)
Biliary drainage							
No (%)	32 (71.1)	29 (74.4)	61 (72.6)	4 (80.0)	5 (100.0)	9 (90.0)	70 (74.5)
Yes (%)	13 (28.9)	10 (25.6)	23 (27.4)	1 (20.0)	0 (0.0)	1 (10.0)	24 (25.5)
Reason of unresectable							
Locally advanced (%)	10 (22.2)	8 (20.5)	18 (21.4)	1 (20.0)	0 (0.0)	1 (10.0)	19 (20.2)
Metastasis (%)	27 (60.0)	26 (66.7)	53 (63.1)	3 (60.0)	5 (100.0)	8 (80.0)	61 (64.9)
Recurrence (%)	8 (17.8)	5 (12.8)	13 (15.5)	1 (20.0)	0 (0.0)	1 (10.0)	14 (14.9)
HBs antigen							
Negative (%)	43 (95.6)	39 (100.0)	82 (97.6)	5 (100.0)	5 (100.0)	10 (100.0)	92 (97.9)
Positive (%)	2 (4.4)	0 (0.0)	2 (2.4)	0 (0.0)	0 (0.0)	0 (0.0)	2 (2.1)
HCV antibody							
Negative (%)	42 (93.3)	38 (97.4)	80 (95.2)	5 (100.0)	5 (100.0)	10 (100.0)	90 (95.7)
Positive (%)	2 (4.4)	1 (2.6)	3 (3.6)	0 (0.0)	0 (0.0)	0 (0.0)	3 (3.2)
Not evaluated (%)	1 (2.2)	0 (0.0)	1 (1.2)	0 (0.0)	0 (0.0)	0 (0.0)	1 (1.1)
Heavy alcohol consumption							
No (%)	39 (86.7)	35 (89.7)	74 (88.1)	3 (60.0)	5 (100.0)	8 (80.0)	82 (87.2)
Yes (%)	6 (13.3)	4 (10.3)	10 (11.9)	2 (40.0)	0 (0.0)	2 (20.0)	12 (12.8)
Child–Pugh score (%)							
5	15 (33.3)	19 (48.7)	34 (40.5)	3 (60.0)	2 (40.0)	5 (50.0)	39 (41.5)
6	15 (33.3)	11 (28.2)	26 (31.0)	0 (0.0)	2 (40.0)	2 (20.0)	28 (29.8)
7	4 (8.9)	3 (7.7)	7 (8.3)	1 (20.0)	0 (0.0)	1 (10.0)	8 (8.5)
8	4 (8.9)	1 (2.6)	5 (6.0)	0 (0.0)	0 (0.0)	0 (0.0)	5 (5.3)
9	2 (4.4)	1 (2.6)	3 (3.6)	0 (0.0)	0 (0.0)	0 (0.0)	3 (3.2)
10	0 (0.0)	1 (2.6)	1 (1.2)	1 (20.0)	0 (0.0)	1 (10.0)	2 (2.1)
Not evaluated	5 (11.1)	3 (7.7)	8 (9.5)	0 (0.0)	1 (20.0)	1 (10.0)	9 (9.6)
White blood cell count (10^3^/μl)							
Median (Range)	6300 (4100–17 570)	6800 (3900–18 000)	6495 (3900–18 000)	6200 (5600–7400)	6100 (4800–7400)	6150 (4800–7400)	6250 (3900–18 000)
Neutrophil count (10^3^/μl)							*N* = 94
Median (Range)	4510 (2430–14 407)	4449 (1770–14 328)	4479.5 (1770–14 407)	4150 (3320–4520)	3772 (3206–5140)	4070 (3206–5140)	4417.5 (3900–18 000)
Hemoglobin (g/dl)							
Median (Range)	12.3 (9.2–15.3)	12.7 (9.6–16.7)	12.5 (9.2–16.7)	12.0 (11.3–14.2)	12.6 (10.3–15.1)	12.3 (10.3–15.1)	12.5 (9.2–16.7)
Platelet count (10^4^/μl)							
Median (Range)	21.7 (13.9–40.9)	20.9 (11.4–43.4)	21.2 (11.4–43.4)	19.1 (12.6–22.9)	17.4 (11.4–28.8)	18.3 (11.4–28.8)	21.0 (11.4–43.4)
Creatinine (mg/dl)							
Median (Range)	0.68 (0.31–1.03)	0.66 (0.40–1.03)	0.68 (0.31–1.03)	0.77 (0.43–0.83)	0.80 (0.50–0.91)	0.78 (0.43–0.91)	0.68 (0.31–1.03)
Total bilirubin (mg/dl)							*N* = 94
Median (Range)	0.70 (0.39–2.70)	0.70 (0.10–2.60)	0.70 (0.10–2.70)	0.70 (0.60–2.75)	0.80 (0.40–1.20)	0.75 (0.40–2.75)	0.7 (0.10–2.75)
Prothrombin time (%) Data available	*n* = 40	*n* = 36	*n* = 76	*n* = 5	*n* = 4	*n* = 9	*n* = 85
Median (Range)	87.5 (34.0–114.0)	90.5 (39.0–117.0)	89.0 (34.0–117.0)	84 (58–88)	98.5 (78.0–105.0)	88.0 (58.0–105.0)	89 (34–117)

GC, gemcitabine plus cisplatin. GS, gemcitabine plus S-1. LC, liver cirrhosis. PS, performance status.

In baseline blood test, white blood cell count, neutrophil count, hemoglobin, creatinine, and prothrombin time were similar between the clinically diagnosed LC and non-LC group, while platelet count was slightly lower in the clinically diagnosed LC group.

### Safety

Table 3 shows the percentage of planned dose administered and the number of courses administered. Percent planned dose administered was slightly lower in the non-LC group, but there were no cases of treatment discontinuation due to AEs in the non-LC group.

**Table 3:** Percent planned dose administered and number of courses administered

	Non-LC			Clinically diagnosed LC			Total
	GC	GS	Total	GC	GS	Total	*n* = 94
	*n* = 45	*n* = 39	*n* = 84	*n* = 5	*n* = 5	*n* = 10	
Percent planned dose administered of gemcitabine (%)							*N* = 94
Median (Range)	81.3 (41.5–101.3)	87.5 (48.9–100.0)	83.3 (41.5–101.3)	75.0 (65.0–100.0)	82.5 (72.3–100.7)	78.8 (65.0–100.7)	83.3 (41.5–101.3)
Percent planned dose administered of S-1 (%)		*n* = 39	*n* = 39				*n* = 44
Median (Range)	—	88.5 (7.1–100.0)	88.5 (71.0–100.0)	—	75.0 (71.7–100.0)	75.0 (71.7–100.0)	87.4 (7.1–100.0)
Percent planned dose administered of cisplatin (%)	*n* = 45		*n* = 45	*n* = 5			*n* = 50
Median (Range)	83.3 (45.5–102.6)	—	83.3 (45.5–102.6)	65.0 (65.0–100.0)	—	65.0 (65.0–100.0)	83.3 (45.5–102.6)
Number of courses administered							
≤8 courses	26	18	44	1	2	3	47
9 courses ≤	19	21	40	4	3	7	47
Reason for discontinuation of treatment							
Continued		2	2	0	0	0	2
Disease progression	34	32	66	5	5	10	76
Adverse event	7	3	10	0	0	0	10
Patient refusal related to adverse events	3	1	4	0	0	0	4
Patient refusal not related to adverse events	1	0	1	0	0	0	1
others	0	1	1	0	0	0	1

The AE profiles in the non-LC and clinically diagnosed LC groups are shown in Table 4. In terms of grade 3 or higher hematological toxicity, the incidence in the non-LC and clinically diagnosed LC groups was as follows: white blood cell decreased (23.8%/20.0%), anemia (15.5%/0%), thrombocytopenia (11.9%/40.0%), and neutropenia (51.2%/60.0%). In the non-LC group, hematological AEs of grade 3 or higher were more frequent in the GC arm (*n* = 45) than in the GS arm (*n* = 39); white blood cell decreased by 31.1%/15.4%; anemia by 24.4%/5.1%; thrombocytopenia by 17.8%/5.1%; and neutropenia by 55.6%/46.2%. In contrast, in the clinically diagnosed LC group, hematological AEs of grade 3 or higher were comparably observed in the GC (*n* = 5) and GS (*n* = 5) arms: white blood cell decreased by 20%/20%; anemia by 0%/0%; thrombocytopenia by 40%/40%; and neutropenia by 60%/60%. One patient in the non-LC group experienced febrile neutropenia after day 15 of the seventh course of GC therapy, died on day 19 (9 days after the final administration), and was judged to have a treatment-related death.

**Table 4:** Summary of adverse events

	Non-LC *n* = 84						Clinically diagnosed LC *n* = 10					
	GC *n* = 45		GS *n* = 39		Total *n* = 84		GC *n* = 5		GS *n* = 5		Total *n* = 10	
	Grade 1–4 *n* (%)	Grade 3–4 *n* (%)	Grade 1–4 *n* (%)	Grade 3–4 *n* (%)	Grade 1–4 *n* (%)	Grade 3–4 *n* (%)	Grade 1–4 *n* (%)	Grade 3–4 *n* (%)	Grade 1–4 *n* (%)	Grade 3–4 *n* (%)	Grade 1–4 *n* (%)	Grade 3–4 *n* (%)
White blood cell decreased	34 (75.6)	14 (31.1)	29 (74.4)	6 (15.4)	63 (75)	20 (23.8)	5 (100)	1 (20)	5 (100)	1 (20)	10 (100)	2 (20)
Anemia	44 (97.8)	11 (24.4)	38 (97.4)	2 (5.1)	82 (97.6)	13 (15.5)	5 (100)	0 (0)	5 (100)	0 (0)	10 (100)	0 (0)
Thrombocytopenia	38 (84.4)	8 (17.8)	31 (79.5)	2 (5.1)	69 (82.1)	10 (11.9)	5 (100)	2 (40)	4 (80)	2 (40)	9 (90)	4 (40)
Neutropenia	37 (82.2)	25 (55.6)	32 (82.1)	18 (46.2)	69 (82.1)	43 (51.2)	5 (100)	3 (60)	5 (100)	3 (60)	100 (100)	6 (60)
Hyperbilirubinemia	12 (26.7)	2 (4.4)	14 (35.9)	3 (7.7)	26 (31)	5 (6)	1 (20)	0 (0)	2 (40)	0 (0)	3 (30)	0 (0)
Elevated ALP	40 (88.9)	7 (15.6)	31 (79.5)	2 (5.1)	71 (84.5)	9 (10.7)	4 (80)	0 (0)	4 (80)	0 (0)	8 (80)	0 (0)
Elevated AST	38 (84.4)	6 (13.3)	35 (89.7)	0 (0)	73 (86.9)	6 (7.1)	2 (40)	0 (0)	4 (80)	1 (20)	6 (60)	1 (20)
Elevated ALT	34 (75.6)	3 (6.7)	30 (76.9)	3 (7.7)	64 (76.2)	6 (7.1)	2 (40)	0 (0)	5 (100)	1 (20)	7 (70)	1 (20)
Increased creatinine	14 (31.1)	1 (2.2)	5 (12.8)	0 (0)	19 (22.6)	1 (1.2)	2 (40)	0 (0)	2 (40)	0 (0)	4 (40)	0 (0)
Febrile neutropenia	1 (2.2)	1 (2.2)	0 (0)	0 (0)	1 (1.2)	1 (1.2)	0 (0)	0 (0)	0 (0)	0 (0)	0 (0)	0 (0)
Diarrhea	10 (22.2)	1 (2.2)	10 (25.6)	1 (2.6)	20 (23.8)	2 (2.4)	0 (0)	0 (0)	0 (0)	0 (0)	0 (0)	0 (0)
Mucositis	6 (13.3)	0 (0)	14 (35.9)	0 (0)	20 (23.8)	0 (0)	0 (0)	0 (0)	1 (20)	0 (0)	1 (10)	0 (0)
Rash	7 (15.6)	0 (0)	10 (25.6)	3 (7.7)	17 (20.2)	3 (3.6)	1 (20)	0 (0)	2 (40)	1 (20)	3 (30)	1 (10)
Biliary tract infection	6 (13.3)	6 (13.3)	3 (7.7)	3 (7.7)	9 (10.7)	9 (10.7)	0 (0)	0 (0)	1 (20)	1 (20)	1 (10)	1 (10)
Fatigue	22 (48.9)	22 (48.9)	13 (33.3)	0 (0)	35 (41.7)	0 (0)	2 (40)	0 (0)	2 (40)	0 (0)	4 (40)	0 (0)
Fever	12 (26.7)	0 (0)	9 (23.1)	1 (2.6)	21 (25)	1 (1.2)	0 (0)	0 (0)	2 (40)	0 (0)	2 (20)	0 (0)
Alopecia	5 (11.1)	− −	5 (12.8)	− −	10 (11.9)	− −	2 (40)	− −	0 (0)	− −	2 (20)	− −
Skin hyperpigmentation	0 (0)	− −	7 (17.9)	− −	7 (8.3)	− −	0 (0)	− −	1 (20)	− −	1 (10)	− −
Nausea	19 (42.2)	1 (2.2)	11 (28.2)	0 (0)	30 (35.7)	1 (1.2)	0 (0)	0 (0)	0 (0)	0 (0)	0 (0)	0 (0)
Vomiting	4 (8.9)	1 (2.2)	3 (7.9)	0 (0)	7 (8.3)	1 (1.2)	1 (20)	0 (0)	0 (0)	0 (0)	1 (10)	0 (0)
Appetite loss	18 (40)	3 (6.7)	13 (33.3)	3 (7.7)	31 (36.9)	6 (7.1)	0 (0)	0 (0)	1 (20)	1 (20)	1 (10)	1 (10)
Dysgeusia	2 (2.2)	− −	6 (15.4)	− −	8 (9.5)	− −	0 (0)	− −	1 (20)	− −	1 (10)	− −
Peripheral motor neuropathy	0 (0)	0 (0)	1 (2.6)	0 (0)	1 (1.2)	0 (0)	0 (0)	0 (0)	0 (0)	0 (0)	0 (0)	0 (0)

LC, liver cirrhosis. GC, gemcitabine plus cisplatin. GS, Gemcitabine plus S-1. LC, liver cirrhosis. ALP, alkaline phosphatase. AST, aspartate transferase. ALT, Alanine Transaminase.

The frequency of non-hematological AEs according to the treatment regimen is shown in Table 5. The frequency of non-hematological AEs of grade 3 or higher was 38.1% (32/84) in the non-LC group and 30% (3/10) in the clinically diagnosed LC group and non-hematological AEs of grade 3 or higher were most frequent in patients treated with GS in the clinically diagnosed LC group (3/5, 60%). Ten patients in the non-LC group discontinued treatment due to AE, but none in the LC group.

**Table 5:** Frequency of non-hematological adverse events in non-LC or clinically diagnosed LC patients by treatment with GC or GS

	Non-LC (*n* = 84)			Clinically diagnosed LC (*n* = 10)		
	GC (*n* = 45)	GS (*n* = 39)	Total (*n* = 84)	GC (*n* = 5)	GS (*n* = 5)	Total (*n* = 10)
All grades	45 (100%)	39 (100%)	84 (100%)	5 (100%)	5 (100%)	10 (100%)
≥ Grade 2	36 (80%)	24 (61.5%)	60 (71.4%)	2 (40%)	4 (80%)	6 (60%)
≥ Grade 3	18 (40%)	14 (35.9%)	32 (38.1%)	0 (0%)	3 (60%)	3 (30%)
≥ Grade 4	1 (2.2%)	0 (0%)	1 (1.2%)	0 (0%)	0 (0%)	0 (0%)

GC, gemcitabine plus cisplatin. GS, gemcitabine plus S-1. LC, liver cirrhosis.

In this study, two patients were categorized as having a Child–Pugh score of 10, but they did not have any AE of grade 3 or higher.

Table 6 shows the frequency of neutropenia and febrile neutropenia according to the presence or absence of CT findings of characteristics of clinically diagnosed LC. Patients with morphological changes in the liver had a higher frequency of grade 3 or higher neutropenia than those without such changes. The frequency of AEs was not necessarily higher in patients with other findings related to clinically diagnosed LC nor did the frequency increase with the number of findings.

**Table 6:** Frequency of neutropenia and febrile neutropenia according to CT imaging of liver cirrhosis

	Neutropenia		Febrile neutropenia	
	≥Grade 3	Grade 4	≥Grade 3	Grade 4
Morphological change				
No	40/81 (49.4%)	8/81 (9.9%)	1/81 (1.2%)	1/81 (1.2%)
Yes	9/13 (69.2%)	3/13 (23.1%)	0/13 (0.0%)	0/13 (0.0%)
Parenchymal abnormalities				
No	46/88 (52.3%)	10/88 (11.4%)	1/88 (1.1%)	1/88 (1.1%)
Yes	3/6 (50.0%)	1/6 (16.7%)	0/6 (0.0%)	0/6 (0.0%)
Blunt edge				
No	39/74 (52.7%)	7/74 (9.5%)	0/74 (0.0%)	0/74 (0.0%)
Yes	10/20 (50.0%)	4/20 (20.0%)	1/20 (5.0%)	1/20 (5.0%)
Irregular surface				
No	45/86 (52.3%)	10/86 (11.6%)	1/86 (1.2%)	1/86 (1.2%)
Yes	4/8 (50.0%)	1/8 (12.5%)	0/8 (0.0%)	0/8 (0.0%)
Varices, collaterals or splenomegaly				
No	42/81 (51.9%)	10/81 (12.3%)	0/81 (0.0%)	0/81 (0.0%)
Yes	7/13 (53.8%)	1/13 (7.7%)	1/13 (7.7%)	1/13 (7.7%)
Ascites				
No	41/75 (54.7%)	11/75 (14.7%)	1/75 (1.3%)	1/75 (1.3%)
Yes	8/19 (42.1%)	0/19 (0.0%)	0/19 (0.0%)	0/19 (0.0%)
Total number of variables				
0	29/51 (56.9%)	7/51 (13.7%)	0/51 (0.0%)	0/51 (0.0%)
1	9/21 (42.9%)	0/21 (0.0%)	0/21 (0.0%)	0/21 (0.0%)
2 or 3	7/18 (38.9%)	3/18 (16.7%)	1/18 (5.6%)	1/18 (5.6%)
4 or 5	4/4 (100.0%)	1/4 (25.0%)	0/4 (0.0%)	0/4 (0.0%)
All^6^	0/0	0/0	0/0	0/0

### Efficacy

#### Prognosis of clinically diagnosed LC compared with non-LC

**Figure 1:**
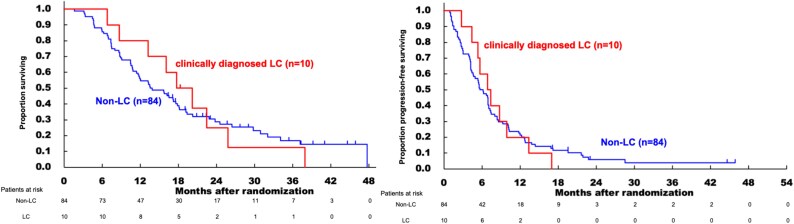
Comparison of overall survival and progression-free survival in clinically diagnosed liver cirrhosis and non- liver cirrhosis groups.

Kaplan–Meier curves for OS and PFS comparing the non-LC and clinically diagnosed LC groups are shown in Fig. 1. The median OS was 13.7 months in the non-LC group and 19.0 months in the clinically diagnosed LC group (HR, 0.969; 95% CI, 0.482**–**1.948). The median PFS was 5.9 months in the non-LC group and 7.1 months in the clinically diagnosed LC group (HR, 0.995; 95% CI, 0.513–1.929). In this study population, there was no significant difference in the prognosis based on the presence or absence of clinically diagnosed LC.

#### Comparing treatment effects of GC and GS therapy according to existence of clinically diagnosed LC

In the non-LC group, 42% (19/45) of patients in the GC arm and 54% (21/39) of patients in the GS arm continued treatment for nine or more cycles, compared to 80% (4/5) and 60% (3/5) in the clinically diagnosed LC group, respectively. Thus, there was no lower rate of continuation of nine or more cycles in the clinically diagnosed LC group.

Table 7 shows the RRs for GC and GS therapies in the non-LC and clinically diagnosed LC groups. There was no consistent trend in the response to GC or GS depending on the presence or absence of clinically diagnosed LC. In both arms, the RR was 30% (24/80) in the non-LC group and 20% (2/10) in the clinically diagnosed LC group.

**Figure 2:**
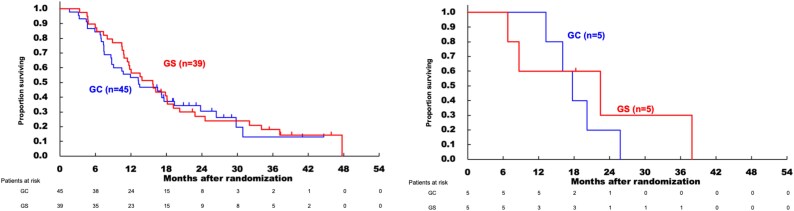
Overall survival comparison of gemcitabine plus cisplatin (GC) and gemcitabine plus S-1 (GS) in non-liver cirrhosis group and clinically diagnosed liver cirrhosis group.

Figure 2 show the OS comparison between patients with GC and GS in the non-LC and clinically diagnosed LC groups. There was no significant difference between the GC and GS arms in terms of OS.

## Discussion

This study showed that clinically diagnosed LC was not a definite risk factor for safety in patients who enrolled in JCOG1113. Although the non-LC group had a slightly lower percentage of the planned dose administered than the clinically diagnosed LC group, there were no cases of treatment discontinuation due to AEs. Therefore, it might be possible to manage AEs and continue treatment with dose modification. Grade 3 or higher AEs were not necessarily more common in the clinically diagnosed LC group than in the non-LC group. Grade 3 or higher thrombocytopenia and neutropenia occurred more frequently in the clinically diagnosed LC group than in the non-LC group, whereas other hematological AEs of grade 3 or higher occurred more frequently in the non-LC group than in the clinically diagnosed LC group. Non-hematological AEs were not more frequent in the clinically diagnosed LC group than in the non-LC group. In terms of efficacy, the presence of clinically diagnosed LC did not necessarily compromise the chemotherapy RR. Additionally, there were no necessarily inferior outcomes in terms of OS and PFS in the clinically diagnosed LC group compared with the non-LC group.

In our study, the Child-Pugh score was not associated with the severity of LC. This is because LC severity could not be assessed simply using the Child–Pugh score in this population. Components of the Child–Pugh score, such as ascites, elevated total bilirubin levels, and decreased albumin levels, could be caused by the cancer itself. However, it is difficult to determine whether the etiology of these events was cancer or LC. It is also possible that the total bilirubin level is elevated because of biliary obstruction or cholangitis in patients with ICC. Indeed, patients after biliary drainage could be enrolled as long as their total bilirubin level ≤3.0 mg/dl in JCOG1113. The Child–Pugh score of patients in the clinically diagnosed LC group was not necessarily higher than that in the non-LC group (Table 2). Additionally, two patients categorized as Child–Pugh C (score of 10), which is usually a contraindication for systemic therapy in HCC, did not have any AE of grade 3 or higher in this study. These facts support the theoretical issues mentioned earlier, and we believe that the Child–Pugh scores of the subjects of this study should not be relied upon. Clinical findings including radiological imaging may be more appropriate for evaluation of LC in patients with advanced ICC.

**Table 7:** Response rate by GC and GS for non-LC and clinically diagnosed LC patients with measurable lesions (*n* = 90).

	Non-LC (*n* = 80)	Clinically diagnosed LC (*n* = 10)
GC (%)	11/43 (25.6)	2/5 (40)
GS (%)	13/37 (35.1)	0/5 (0.0)
Total (%)	24/80 (30.0)	2/10 (20.0)

GC, gemcitabine plus cisplatin. GS, gemcitabine plus S-1. LC, liver cirrhosis.

The reason why this study did not find a clear difference between clinically diagnosed LC and non-LC may be related to the limitations of this study. The number of patients diagnosed with the clinically diagnosed LC in this study was small. In addition, the diagnosis of LC may have been somewhat subjective because it was clinically diagnosed by physicians based on radiological findings, clinical history, laboratory data, and physical findings without histological diagnosis. Other points that should be interpreted with caution in this study is that patients classified as clinically diagnosed with LC still had sufficient organ function to meet the enrollment criteria for a clinical trial. Patients with platelets <100 × 10^9^/l, bilirubin >3 mg/dl, moderate or severe ascites, or psychiatric symptoms including hepatic encephalopathy were excluded. These findings may not reflect the actual clinical conditions of the entire population of patients with LC. These may have influenced the lack of a clear difference between the clinically diagnosed LC and non- LC groups in this study.

Another limitation is the breakdown of etiology. In the clinically diagnosed LC group, all patients tested negative for both HBV and HCV, and 20% had a history of heavy alcohol consumption. A certain number of the remaining patients with LC may have nonalcoholic steatohepatitis. However, we do not have enough information to diagnose nonalcoholic steatohepatitis; therefore, the specific percentage is unknown. However, this breakdown is not typical of the etiology of LC in Japan. This may be due to the small sample size. Alternatively, morphological changes in the liver caused by cancer, such as portal vein stenosis, could have contributed to the unusual breakdown of etiology.

We conclude that chemotherapy may be indicated in patients with advanced ICC and good systemic condition (eligible for clinical trials) even if LC is clinically suspected. The appropriateness of chemotherapy in patients in daily practice with LC levels that would disqualify them from clinical trials (platelets <100 × 10^9^/l, bilirubin >3 mg/dl, moderate or severe ascites, or psychiatric symptoms, including hepatic encephalopathy) remains a clinical question to be resolved in the future.

## Data Availability

The authors confirm that data supporting the findings of this study are available within this article.

## Notes

Conflict of interest: Dr. Okada, Dr. Suzuki, declare no competing interests. Dr. Morizane reports grants from Eisai, Yakult Honsha, ONO Pharmaceutical, Taiho Pharmaceutical, J-Pharma, AstraZeneca, Merck biopharma, Daiichi Sankyo, HITACHI, Boehringer Ingelheim, Chugai, Labcorp, and reports payment or honoraria for lectures, presentations, speakers bureaus, manuscript writing or educational events from Yakult Honsha, Teijin Pharma, Taiho Pharmaceutical, Eisai, MSD K.K., AstraZeneca, SERVIER, Guardant, Chugai, Myriad. Dr. Ogawa reports supports from Japan Agency for Medical Research and Development, and Ministry of Health, Labour and Welfare, Japan. Dr. Y. Sano reports support from National Cancer Center research fund, Grants from AstraZeneca, Ono Pharmaceutical Co., Ltd, Chugai Pharmaceutical Co., Ltd, Takeda Pharmaceutical Company Limited. Dr. Imaoka reports grants from Ono Pharmaceutical, Servier, Novartis, Boehringer Ingelheim, and payment or honoraria and others from Servier, Yakult Honsha, Boston Scientific, Kaneka Medix, Medico’s Hirata, SB KAWASUMI LABORATORIES, AstraZeneca, Novartis and payment for expert testimony from Kaneka Medix, and Participation on a Data Safety Monitoring Board or Advisory Board from Servier. Dr. Kobayashi reports payment or honoraria and others from AstraZeneca plc., MSD Co., Ltd., and Taiho Pharmaceutical. Dr. Ikeda reports Consulting fees from AstraZeneca, Boehringer Ingelheim, MSD and payment or honoraria and others from AstraZeneca, Guardant Health Japan, Incyte Biosciences Japan, Taiho, and research funding from AstraZeneca, Boehringer Ingelheim, Eisai, J-Pharma, Merck biopharma, Ono, Lilly Japan. Dr. Okano reports honoraria from Taiho Pharmaceutical, Eisai, Bayer Yakuhin, Chugai Pharmaceutical, Ono Pharmaceutical, Daiichi Sankyo, AstraZeneca, MSD, Incyte, Nihon Servier. Dr. Miwa declares no competing interest. Dr. Todaka reports Payment for expert testimony from Ono Pharmaceutical, Nihon Servier, Yakult Honsha, Teijin Pharma Limited, Taiho Pharmaceutical, AstraZeneca K.K., Novartis Pharma K.K, Bristol-Myers Squibb K.K.

Dr Shimizu reports grants from AstraZeneca, Incyte Corporation and Delta-Fly Pharma, and Nihon Servier. Dr. Mizuno reports supports from Ministry of Health, Labour and Welfare, Japan, and Japan Agency for Medical Research and Development (AMED), and grants from Novartis, MSD, Incyte, Ono Pharmaceutical, Seagen, Boehringer Ingelheim, Pfizer, AstraZeneca, and honoraria and others from Yakult Honsha, AstraZeneca, Novartis, FUJIFILM Toyama Chemical, Taiho Pharmaceutical, Miyarisan pharmaceutical, SERVIER, and Participation on a Data Safety Monitoring Board or Advisory Board of MSD and Boehringer Ingelheim. Dr. Satoi reports grants from Nihon Servier, and Boston Scientific. Dr. K. Sano, Dr. Tobimatsu, Dr. Katanuma, Dr. Masutomi declare no competing interests. Dr. Okusaka reports Japan Agency for Medical Research and Development (AMED) and National Cancer Center research fund, and grants from Chugai Pharmaceutical, Eisai, Novartis Pharma, Bristol-Myers Squibb Company, AstraZeneca, MSD, Chiome Bioscience, Syneos, Incyte jp, SYSMEX, and Consulting fees from AstraZeneca, Eisai, Ono Pharmaceutica, FUJIFILM Toyama Chemical, Daiichi Sankyo, Dainippon Sumitomo Pharma, Nihon Servier, Chugai Pharmaceutical, Novartis Pharma. Dr. Ozaka reports Payment or honoraria for lectures and others from Taiho, Ono, Yakult, Servier, MSD, Pfizer, Bayer, and Novartis. Dr. Ueno reports grants from Taiho Pharmaceutical Co., Ltd., AstraZeneca, K.K, MSD K.K, Nihon Servier Co., Ltd., Ono Pharmaceutical Co., Ltd., Incyte Biosciences Japan GK, Chugai Pharmaceutical Co., Ltd., Boehringer Ingelheim GmbH, Eisai Co., Ltd., Novartis Pharma K.K, Astellas Pharma Inc., J-pharma Co., Ltd., DFP (Delta Fly Pharma), Inc., Novocure GmbH, and Chiome Bioscience Inc., and reports personal fees and other from Taiho Pharmaceutical Co., Ltd., AstraZeneca, K.K, Yakult Honsha Co., Ltd., MSD K.K, Nihon Servier Co., Ltd., Ono Pharmaceutical Co., Ltd., Incyte Biosciences Japan GK, Chugai Pharmaceutical Co., Ltd., Boehringer Ingelheim GmbH, J-pharma Co., Ltd., Daiichi Sankyo Co., Ltd., Eisai Co., Ltd., Takeda Pharmaceutical Co., Ltd., and Novartis Pharma K.K.

## References

[1] Sung H, Ferlay J, Siegel RL. et al. Global cancer statistics 2020: GLOBOCAN estimates of incidence and mortality worldwide for 36 cancers in 185 countries. CA Cancer J Clin 2021;71:209–49. 10.3322/caac.21660. 33538338

[2] Palmer WC, Patel T. Are common factors involved in the pathogenesis of primary liver cancers? A meta-analysis of risk factors for intrahepatic cholangiocarcinoma. J Hepatol 2012;57:69–76. 10.1016/j.jhep.2012.02.022. 22420979 PMC3804834

[3] Shaib YH, el-Serag HB, Davila JA, Morgan R, McGlynn KA. Risk factors of intrahepatic cholangiocarcinoma in the United States: a case-control study. Gastroenterology 2005;128:620–6. 10.1053/j.gastro.2004.12.048. 15765398

[4] Welzel TM, Graubard BI, el–Serag HB. et al. Risk factors for intrahepatic and extrahepatic cholangiocarcinoma in the United States: a population-based case-control study. Clin Gastroenterol Hepatol 2007;5:1221–8. 10.1016/j.cgh.2007.05.020. 17689296 PMC2083573

[5] Pinter M, Trauner M, Peck-Radosavljevic M, Sieghart W. Cancer and liver cirrhosis: implications on prognosis and management. ESMO Open 2016;1:e000042. 10.1136/esmoopen-2016-000042. 27843598 PMC5070280

[6] Clements O, Eliahoo J, Kim JU, Taylor-Robinson SD, Khan SA. Risk factors for intrahepatic and extrahepatic cholangiocarcinoma: a systematic review and meta-analysis. J Hepatol 2020;72:95–103. 10.1016/j.jhep.2019.09.007. 31536748

[7] Kam AE, Masood A, Shroff RT. Current and emerging therapies for advanced biliary tract cancers. Lancet Gastroenterol Hepatol 2021;6:956–69. 10.1016/S2468-1253(21)00171-0. 34626563

[8] Banales JM, Marin JJG, Lamarca A. et al. Cholangiocarcinoma 2020: The next horizon in mechanisms and management. Nat Rev Gastroenterol Hepatol 2020;17:557–88. 10.1038/s41575-020-0310-z. 32606456 PMC7447603

[9] Khan SA, Toledano MB, Taylor-Robinson SD. Epidemiology, risk factors, and pathogenesis of cholangiocarcinoma. HPB (Oxford) 2008;10:77–82. 10.1080/13651820801992641. 18773060 PMC2504381

[10] Kudo M, Izumi N, Kokudo N. et al. Report of the 22nd nationwide follow-up survey of primary liver cancer in Japan (2012-2013). Hepatology research: the official journal of the Japan Society of Hepatology 2022;52:5–66. 10.1111/hepr.13675. 34050584

[11] Valle J, Wasan H, Palmer DH. et al. Cisplatin plus gemcitabine versus gemcitabine for biliary tract cancer. N Engl J Med 2010;362:1273–81. 10.1056/NEJMoa0908721. 20375404

[12] Morizane C, Okusaka T, Mizusawa J. et al. Combination gemcitabine plus S-1 versus gemcitabine plus cisplatin for advanced/recurrent biliary tract cancer: The FUGA-BT (JCOG1113) randomized phase III clinical trial. Ann Oncol 2019;30:1950–8. 10.1093/annonc/mdz402. 31566666

[13] Bosilkovska M, Walder B, Besson M, Daali Y, Desmeules J. Analgesics in patients with hepatic impairment: Pharmacology and clinical implications. Drugs 2012;72:1645–69. 10.2165/11635500-000000000-00000. 22867045

[14] Verbeeck RK . Pharmacokinetics and dosage adjustment in patients with hepatic dysfunction. Eur J Clin Pharmacol 2008;64:1147–61. 10.1007/s00228-008-0553-z. 18762933

[15] García-Cortés M, García-García A. Management of Pharmacologic Adverse Effects in advanced liver disease. Clin Drug Investig 2022;42:33–8. 10.1007/s40261-022-01150-w. PMC9205818 35522395

[16] Qamar AA, Grace ND, Groszmann RJ. et al. Incidence, prevalence, and clinical significance of abnormal hematologic indices in compensated cirrhosis. Clin Gastroenterol Hepatol 2009;7:689–95. 10.1016/j.cgh.2009.02.021. 19281860 PMC4545534

[17] LiverTox: Clinical and research information on drug-induced liver injury. Bethesda (MD): National Institute of Diabetes and Digestive and Kidney Diseases, 2012.

[18] Teusink AC, Hall PD. Toxicities of gemcitabine in patients with severe hepatic dysfunction. Ann Pharmacother 2010;44:750–4. 10.1345/aph.1M587. 20233917

[19] Joerger M, Huitema AD, Koeberle D. et al. Safety and pharmacology of gemcitabine and capecitabine in patients with advanced pancreatico-biliary cancer and hepatic dysfunction. Cancer Chemother Pharmacol 2014;73:113–24. 10.1007/s00280-013-2327-2. 24166106

[20] Shibata T, Ebata T, Fujita K. et al. Optimal dose of gemcitabine for the treatment of biliary tract or pancreatic cancer in patients with liver dysfunction. Cancer Sci 2016;107:168–72. 10.1111/cas.12851. 26595259 PMC4768397

[21] Kudo M, Zheng RQ, Kim SR. et al. Diagnostic accuracy of imaging for liver cirrhosis compared to histologically proven liver cirrhosis. A Multicenter Collaborative Study Intervirology 2008;51:17–26. 10.1159/000122595. 18544944

[22] Huber A, Ebner L, Heverhagen JT, Christe A. State-of-the-art imaging of liver fibrosis and cirrhosis: A comprehensive review of current applications and future perspectives. Eur J Radiol Open 2015;2:90–100. 10.1016/j.ejro.2015.05.002. 26937441 PMC4750581

[23] Pugh RN, Murray-Lyon IM, Dawson JL, Pietroni MC, Williams R. Transection of the oesophagus for bleeding oesophageal varices. Br J Surg 1973;60:646–9. 10.1002/bjs.1800600817. 4541913

[24] Gluud C, Henriksen JH, Nielsen G. Prognostic indicators in alcoholic cirrhotic men. Hepatology 1988;8:222–7. 10.1002/hep.1840080205. 3258578

